# Dietary Behaviour of Pregnant Women in Ethiopia: The Missing Aspect of Care

**DOI:** 10.3390/nu16193227

**Published:** 2024-09-24

**Authors:** Simegn Kassa Alamirew, Stefanie Lemke, Bernhard Freyer, Barbara Stadlmayr

**Affiliations:** 1 Institute of Development Research (IDR), BOKU University, 1190 Vienna, Austria; simegn.alamirew@students.boku.ac.at (S.K.A.); stefanie.lemke@boku.ac.at (S.L.); 2 Institute of Organic Farming, BOKU University, 1190 Vienna, Austria; bernhard.freyer@boku.ac.at

**Keywords:** dietary behaviour, diet, pregnant women, Ethiopia, care

## Abstract

**Background**: Nutrition and adequate dietary intake during pregnancy strongly influence the health and well-being of the mother, as well as the physical and cognitive development of the unborn child. While previous studies have documented factors associated with the dietary behaviour of pregnant women in Ethiopia, a comprehensive overview is missing. **Objective**: The aim of this study was to close this research gap. **Methodology**: We conducted a mapping review, including 37 studies published between 2000 and 2022 in our analysis. Dietary behaviour refers to all phenomena related to food choice, eating behaviour and dietary intake. We used an innovative approach by integrating a socio-ecological framework with UNICEF’s conceptual framework on maternal and child nutrition, which specifies multidimensional individual, underlying and enabling determinants associated with the nutritional status of women. Importantly, we integrated a focus on care for women and healthy environments. **Results**: A total of 68 factors were identified as influencing the dietary behaviour of pregnant women, with a focus on the intra- (31/68) and interpersonal (21/68) levels, while factors at the community (11/68) and the institutional levels (5/68) were scarce. Few studies investigated socio-cultural aspects, such as gender roles, decision-making power and workload of women, psychological factors and eating practices related to food taboos. None of the studies explored the influence of resources at the institutional level. **Conclusions**: This attests that the focus in maternal nutrition is still placed on the individual responsibility of women, instead of addressing the structural conditions that would enable women to access resources such as land, education and nutrition information.

## 1. Introduction

Nutrition during pregnancy strongly influences the well-being of the mother, as well as the physical and cognitive development of the foetus [1,2]. Pregnant women need additional micronutrients and energy to meet their own metabolic and physiological demands, as well as those of their unborn children [3,4]. Even though progress has been made globally, maternal malnutrition remains high across some regions in South Central and South East Asia and Sub-Saharan Africa [5]. In Sub-Saharan Africa, malnutrition, under-nutrition and anaemia are among the most prominent public health problems of pregnant women [6]. The prevalence of anaemia among women of reproductive age (WRA) in Africa ranges from 22% in Ethiopia to 72.5% in Burkina Faso [7]. A recent systematic review of the literature on four Sub-Saharan African countries reported the prevalences of the following micronutrient deficiencies among pregnant women: iron deficiency anaemia ranged from 9% in Ethiopia to 47% in Nigeria; vitamin A deficiency ranged from 31% in Ethiopia to 48% in Nigeria; and iodine deficiency was 87% in Ethiopia [6]. In Ethiopia, in addition to the high prevalence of iodine deficiency, the prevalence of under-nutrition among pregnant women ranged from 17.4% to 47.9% in different regions [8,9], while the overall prevalence of different types of anaemia among pregnant women ranged from 8% to 52% [10,11]. The wide range in the prevalence of anaemia might be due to the different socio-demographic and health conditions of pregnant women, as well as differences in study design, such as sampling and assessment methods.

The United Nations Children’s Fund (UNICEF)’s conceptual framework on maternal and child nutrition categorises multidimensional determinants at three levels. These include (1) immediate determinants, which are related to individual adequate and nutritious diets, as well as good care; (2) underlying determinants at the household and community levels, related to access to nutrient-rich foods, adequate food preparation, food consumption and hygiene practices, as well as adequate nutrition, health, sanitation, education and social protection services, including a healthy food and living environment; (3) enabling determinants at the macro level, related to good governance in terms of political, financial, social, public and private sector institutions and actions, as well as adequate environmental, financial, social and human resources, and further gender, cultural and social norms that enhance adequate nutrition for women and children [12].

The biological and developmental path of a child is embedded in the context of a nurturing relationship, with the mother–child dyad [13] showing the significance of care for women at different levels for themselves and for the child. Quality of care, particularly for women and children, is increasingly recognised internationally as a critical aspect of the unfinished maternal and new-born health agenda [14]. The WHO–UNICEF Lancet commission, in 2020, launched a report entitled “A Future for the World’s Children”, giving priority to care for children 0–18 years of age in terms of health and well-being as one of the landmarks in achieving the Sustainable Development Goals (SDGs) [13]. The report further puts emphasis on care for women, regarding their right to nutrition and a healthy environment, as major aspects in the promotion of the good health and well-being of children [13]. Therefore, care for children is directly interlinked with care for women.

In 2016, the government of Ethiopia launched the “Seqota” Declaration, with the goal of eliminating all forms of malnutrition among children under two years of age by 2030, with programmes targeted at improving the health and nutritional statuses of women, children under two years of age and adolescent girls [15]. As the health and well-being of a child begins during pregnancy, and even before conception, understanding the enablers and barriers in the dietary behaviour of pregnant women and the related issues of care and a healthy environment is fundamental for achieving adequate nutrition and well-being for women and their children.

This mapping review used an innovative approach by integrating the UNICEF framework with the socio-ecological framework to categorise the enablers and barriers in the dietary behaviour of pregnant women in Ethiopia at different levels. According to Stok et al. (2018), dietary behaviour refers to all phenomena related to food choice, eating habits and dietary intake [16].

Several studies have investigated the determinants of dietary behaviours among women of reproductive age in different contexts in Africa. A systematic review explored dietary and physical activity behaviours in urban Sub-Saharan Africa. One study applied an urban food environment framework in the creation of healthy nutrition policy and interventions; a qualitative systematic review explored the enablers and barriers of healthy dietary behaviour in Ethiopia based on a socio-ecological model; and a study investigates dietary practices and associated factors among pregnant women in Northwest Ethiopia [17,18,19,20]. Other systematic mapping reviews investigated the factors influencing dietary behaviour among women and men 18–70 years of age, as well as female and male adolescents aged 11–17 years of age, in urban environments in Africa [21] and pregnant women’s diet in Ethiopia, focusing on the factors associated with dietary diversity as a proxy for diet quality [22]. However, these studies do not provide a comprehensive overview of the dietary behaviour of pregnant women. For example, a qualitative systematic review [19] involving heterogeneous participants and did not comprehensively include quantitative, qualitative and mixed-methods studies to get more detailed information about enablers and barriers of dietary behaviour, while the quantitative systematic review [22] on pregnant women did not consider the broader range of dietary behaviour beyond dietary intake.

The aim of this study is to close this research gap by identifying and synthesising enablers and barriers of the dietary behaviour of pregnant women in Ethiopia and related issues of care for women and a healthy environment.

## 2. Materials and Methods

**Figure 1:**
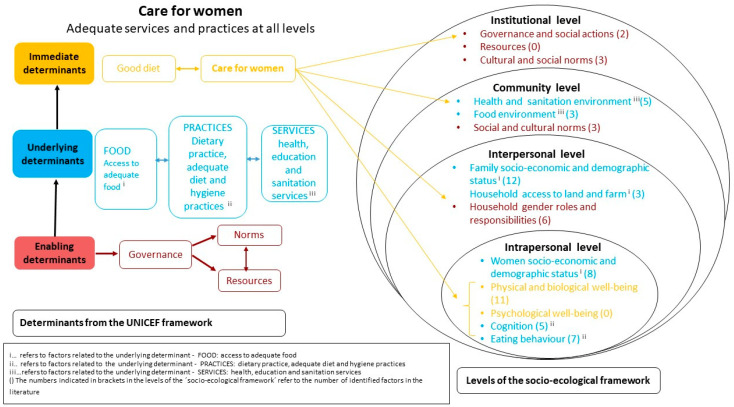
Factors influencing the dietary behaviour of pregnant women according to the socio-ecological framework, integrated with the UNICEF 2020 conceptual framework.

We conducted a mapping review, which is a systematic approach to identifying, describing and cataloguing the evidence and evidence gaps for a broader topic area [23,24]. This also provided insights into how the socio-ecological model had been applied previously in the context of dietary behaviour. Care for women and a healthy environment, which are fundamental factors in the context of dietary behaviour and adequate nutrition, have so far been missing in the socio-ecological framework. This led us to integrate and adapt this model with elements from the UNICEF framework [23,24]. The next section briefly introduces the origins and characteristics of both frameworks and how we adapted them to our specific research context. The conceptual framing and integration of both frameworks is briefly outlined and presented in more detail in Section 3.2 “Adaptation of the Socio-Ecological Framework and Integration with UNICEF Framework Highlighting Care for Women” (see also Figure 1).

### 2.1. Conceptual Framework

To understand the multidimensional factors in the dietary behaviour of pregnant women, we drew on the socio-ecological conceptual framework (The Ecology of Human Development). This framework was originally developed by psychologist Urie Bronfenbrenner in the late 1970s to show that individuals are affected by a different range of environmental systems with which individuals interact [25]. This model has been used widely as a framework to understand dietary behaviour and for illustrating the close interlinkages of individual, social, cultural, physical and environmental factors and how they influence each other [17,19,21] (see Figure 1).

We adapted the categories at each level of the socio-ecological framework to our specific context from Yiga et al. (2021) [17], who applied the model to understand the determinants of dietary and physical activity behaviours among women of reproductive age in urban Sub-Saharan African contexts. Our adapted framework includes the intrapersonal (micro-)level, the interpersonal (household) level, the community level and the institutional (macro-)level. Using a novel approach, we integrated key elements from UNICEF’s conceptual framework on maternal and child nutrition in order to identify and categorise important complementary factors of dietary behaviour of pregnant women, as was explained earlier [12].

The UNICEF conceptual framework on maternal and child nutrition was originally designed in 1991 to provide a basis for the assessment, analysis and actions towards understand child and maternal malnutrition and to improve child and maternal nutrition and development [26]. The framework includes basic (macrolevel), underlying (household/community level) and immediate (microlevel) causes of child and maternal malnutrition. In the original framework of 1991, the focus was on barriers and causes of malnutrition rather than on the drivers or enablers of healthy nutrition for children and women. Moreover, the subcategories at the basic, underlying and immediate levels were framed in a broad manner and were specified and refined in the recent framework [12], with the latter placing more emphasis on the enabling determinants of good maternal and child nutrition. This revised UNICEF framework further shifts the perspective/paradigm towards women’s (and children’s) right to nutrition, moving away from the dominant needs-based approach that framed women and children as victims and not as rights holders.

According to the UNICEF framework, good care is the result of adequate practices (e.g., dietary practices, referring to adequate food preparation, food consumption and hygiene practices) and adequate services (e.g., adequate nutrition, health, education, sanitation and social protection services, as well as healthy food environments) that support good diets [12]. To place more emphasis on the importance of good care for pregnant women, we therefore added “care for women” as a new subcategory at all levels of the socio-ecological framework (see Figure 1).

We further adapted the outcome variable ‘’dietary behaviour’’ based on an article by Stok et al. (2018) [16]. The authors define dietary behaviour as an ‘‘overall umbrella term referring to all phenomena related to food choice, eating behaviour, and dietary intake/nutrition’’ [16]. According to Stok et al. (2018), food choice refers to the behaviours and other factors occurring before food is actually consumed, such as preference, frequency of purchase, food preparation and intention to choose, buy, or consume food; eating behaviour refers to outcomes related to the actual act of consumption, such as eating habits and eating occasions; dietary intake/nutrition refers to outcomes of the actual eating occasion of food consumption, including the quality, quantity and diversity of food consumed [16]. In our review, we identified the following dietary behaviours of pregnant women: (1) dietary practice (i.e., dietary habits including meal frequency, dietary diversity and nutrition knowledge); (2) dietary diversity (quantitative indicator to assess adequate or inadequate micronutrient intake); (3) nutrition practices; (4) dietary/nutrition knowledge and information; (5) food consumption score (i.e., frequency of food consumed); (6) taboo food practices; (7) food aversion (i.e., dislike or avoidance of particular food).

### 2.2. Inclusion and Exclusion Criteria

We selected articles applying the following inclusion and exclusion criteria (Table 1).

### 2.3. Search Strategy

For this review, we conducted three rounds of literature searches. The first search included pregnant women, focusing on quantitative studies via Scopus, PubMed and Google Scholar. The second search focused on qualitative studies using Scopus and PubMed. However, as the first search was completed by April 2022, the literature was updated for a third time by the end of 2022 to expand the range of available studies. The following keywords were used to search for relevant studies with “AND/OR” conjunctions: “dietary practice, dietary diversity, dietary behaviour, eating habit, food intake, diet, food taboo, food consumption, diet quality, nutrition, pregnant women, mother, women, female, caregiver, Ethiopia care for mother, household responsibility, household task, gender role, workload, household decision”.

### 2.4. Data Extraction

Data were extracted in Excel by the first author (SK.A.) using a data extraction spread sheet and (20%) checked by one of the co-authors (B.S.). The data extracted included the characteristics of the selected studies, such as the authors’ names, title, year, setting (urban/rural), region, aim of the study, study design, sample size and age, as well as the outcomes of the dietary behaviour, description of dietary behaviour, and factors influencing dietary behaviour.

### 2.5. Data Synthesis

The synthesis was carried out in two stages. In the first stage, all factors identified in the selected studies that influence the dietary behaviour of pregnant women were extracted. In the second stage the factors were sorted and structured into clusters, according to our adapted socio-ecological framework.

## 3. Results

### 3.1. Characteristics of Included Studies

**Figure 2:**
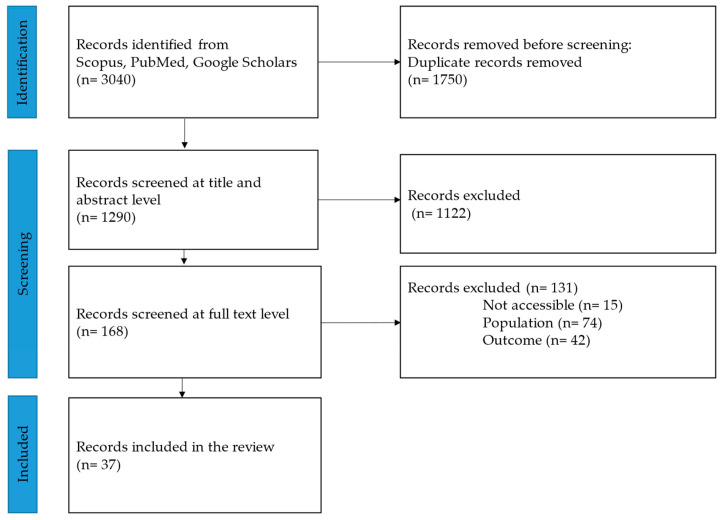
Flowchart of the search process.

In total, 37 studies met the inclusion criteria and were included in the review. Figure 2 presents the study selection process and related PRISMA flowchart. Table 2A,B summarise the characteristics of the included studies.

The included studies (*n* = 37) were conducted in six regions and two federal states in Ethiopia. Most of the studies were conducted in the Oromia region (*n* = 11), followed by Southern Nations, Nationalities and People Region (SNNPR) (*n* = 9) and Amhara Region (*n* = 8). Three studies were conducted in Addis Ababa capital city of Ethiopia, two studies each in Tigray and Afar and one study each in Somali Region and the federal city of Dire Dawa.

The majority were quantitative studies (76%; *n* = 28), six studies had mixed-methods designs (i.e., quantitative and qualitative) and three were qualitative studies. With regard to the urban/rural context, 30% of the studies were conducted in urban areas, 32% in rural areas and 38% in both rural and urban areas. All studies adopted a cross-sectional study design. Most quantitative and mixed-methods studies used structured interviews and a few studies used structured and semi-structured interviews for data collection.

### 3.2. Adaptation of the Socio-Ecological Framework and Integration with UNICEF’s Framework Highlighting Care for Women

We adapted the initial socio-ecological framework from Yiga et al. (2021) [17] by integrating the category “care for women” from the UNICEF framework [12] at each level (intrapersonal, interpersonal, community and institutional levels), as described in Section 2 (Materials and Methods) and illustrated in Figure 1. Moreover, we adapted sublevels within the intrapersonal, interpersonal, community and institutional levels as outlined by Yiga et al. (2021). We addressed this by integrating new sublevels based on the UNICEF framework [12] and by adapting sublevels based on our data (i.e., newly identified factors), after discussion among the authors. For example, we added the sublevel “cognition” and the sublevel “physical and biological well-being” to the intrapersonal level. At the interpersonal level, we added the sublevels “household access to land” and “household gender roles and responsibilities”. At the community level, we added the sublevel “health and sanitation environment” from the UNICEF framework. At the institutional level we added the sublevels “governance” and “resources” based on the UNICEF framework. More detailed information on each level is outlined below. Overall, the framework (see Figure 1) shows the diversity of factors across the different levels of influence, highlighting the need for multiple, context-specific approaches to improve the dietary behaviour of pregnant women.

Intrapersonal level: The intrapersonal (individual/micro-) level was integrated with the immediate and underlying levels of the UNICEF framework related to care for women in terms of diet, practice, health, and well-being. In this review, care for women at the intrapersonal level indicates pregnant women’s own actions and behaviours that enhance their personal healthy dietary behaviour. It is important to note that these actions and behaviour depend, to a large extent, on the available resources at the macro- and community levels, as well as on norms that determine access to resources and decision making for women. In the UNICEF framework, these are shown as enabling determinants, consisting of (1) sufficient resources, including environmental, financial, social and human resources, that enable women’s (and children’s) right to nutrition (reflected in the socio-ecological framework at the institutional level), and (2) norms, referring to positive cultural norms and actions, which enable women’s (and children’s) right to nutrition. Sufficient resources are partly reflected in the sublevel economic status of women, as described below, and are also relevant at the interpersonal/household (socio-economic statuses of household members) and community levels (food environment). The intrapersonal level is divided into five sublevels. The first sublevel is the socio-economic and demographic status of women, which includes factors such as age, marital status, education, occupation and women’s monthly incomes. In our adapted framework, four sublevels relate to care for women, as follows: (1) physical and biological well-being, such as the health condition of pregnant women, gestational age of pregnancy, gravida (number of pregnancies), nutrition status, and history of illness; (2) psychological well-being; (3) cognition, including attitudes towards preparing nutritious food and consumption and perception of balanced diet/nutrition, as well as dietary/nutrition knowledge and information held by pregnant women; and (4) eating behaviour of pregnant women, which includes meal frequency, meal skipping, food aversion, additional meals apart from their usual and psychological factors, such as emotional satisfaction.

Interpersonal level: The interpersonal (household) level was integrated with the underlying level of the UNICEF framework related to access to food, care for women in terms of practices such as household roles and responsibilities. In our adapted framework, this level included the following three sublevels: (1) socio-economic and demographic statuses of household members; (2) household access to land and farming; and (3) gender roles and responsibilities. Care for women at this level refers to the contribution of household members to supporting and encouraging pregnant women to engage in healthy dietary behaviours. In Ethiopia, taking care of the family and all domestic chores are regarded as the responsibility of women, also during pregnancy [59]. We identified factors related to roles and contributions of household members that influence dietary behaviour of pregnant women. These include women’s decision on food preparation and food allocation; emotional support provided by husbands; shared responsibilities within the household to avoid high workloads being placed on women;. The socio-economic and -demographic statuses of household members include factors that influence the dietary behaviour of pregnant women, such as the husband’s level of education, household wealth index, household monthly income, and household size. Regarding access to land and farming, factors associated with the dietary behaviour of pregnant women are, among others, land ownership, having a home garden, and crop and vegetable production for household consumption.

Community level: The community level was integrated with specific aspects from the underlying level of the UNICEF framework related to the food environment, adequate services, such as health and sanitation environment, particularly health services in terms of care for women. Further, the category norms from the enabling level of the UNICEF framework was also integrated here, as social and cultural norms at the community level determine food practices, for example, during religious and cultural events, further impacting individual practices. Care for women at the sublevel of the health and sanitation environment relates to adequate health services designed for pregnant women, such as ANC (antenatal counselling), which is an indicator of access to and use of health care during pregnancy, and PNC (postnatal counselling), which is an indicator of access to and use of health care after delivery, as well as nutrition information and health education during pregnancy. Sanitation refers to adequate facilities that support pregnant women, such as hygiene and sanitation education during pregnancy, suitable and accessible latrines for pregnant women, particularly in rural areas, and other facilities. The sublevel food environment includes aspects related to a conducive environment for food preparation and consumption, availability and accessibility of agricultural production, such as market availability, and production and seasonality of production. Social and cultural norms influence food practices in general. For example, because of cultural beliefs certain foods are said to be taboo for women during pregnancy (as well as in other stages, such as the menstrual cycle), impacting food practices at the interpersonal and intrapersonal levels.

Institutional level: The institutional (macro-)level was integrated with specific aspects of the enabling level from the UNICEF framework, for example, governance related to public and social actions, as well as factors related to care for women, such as access to resources and cultural and social norms. Care for women at this level refers to good governance with regard to political, financial, social, public and private sector actions that enhance the right to adequate and healthy diets for pregnant women. For example, in previous years the Ethiopian government has provided financial support for pregnant or lactating women and infant children targeting food-insecure households, such as the Productive Safety Net Program (PSNP). In addition to food aid, other support is provided to access adequate food. Government, NGOs and public organisations also support women’s economic and social empowerment, such as Village Savings and Loan Associations (VSLAs) in Ethiopia. UN Women in Ethiopia works on economically empowering women through a programme called Women’s Cooperative Revolving Fund, which facilitates loans and savings to involve women in micro-investments and provide psychological support. Sublevel resources refer to sufficient environmental, financial, social and human resources. Sublevel social and cultural norms refer to gender and other social norms and actions with regard to the dietary behaviour of pregnant women, for example, religious institutions and related norms, such as religious fasting practices, and ethnicity and related norms affecting the dietary behaviour of pregnant women.

### 3.3. Factors Influencing the Dietary Behaviour of Pregnant Women

In this section, we present the identified factors, categorised according to the conceptual framework described above and their importance in the dietary behaviour of pregnant women. The results are presented narratively for each factor. Table 2A,B provide an overview of the evidence.

A total of 68 factors were identified that influence the dietary behaviour of pregnant women in Ethiopia. All factors were mapped according to our adjusted conceptual framework (see Figure 1). Most factors (*n* = 31/68) were identified at the intrapersonal level, followed by the interpersonal level (*n* = 21/68). The smallest numbers of factors were identified at the community level (*n* = 11/68) and at the institutional level (*n* = 5/68) (see Table 2A,B.

#### 3.3.1. Intrapersonal Level

In total, we found 31 intrapersonal factors that influence the dietary behaviour of pregnant women, which we divided into the following four sublevels: socio-economic and demographic statuses of women (*n* = 8/31), physical and biological well-being (*n* = 12/31), cognition (*n* = 4/31) and eating behaviour (*n* = 7/31).

Socio-economic and demographic status

At this sublevel, we found the following eight factors: educational status, age of pregnant women, occupation, marital status, years of marriage, own monthly income, having a mobile phone and having a savings account. At this level, among the eight factors discussed in this section, we present the three most studied factors, as follows: educational status, age and occupation of pregnant women. Women’s own monthly income, which has rarely been studied but a most relevant factor, is included in the narrative synthesis..

Educational status: This was the most studied factor at the sublevel of socio-economic and -demographic statuses, reported in 19 quantitative studies [4,8,27,28,29,30,31,32,38,39,40,42,44,45,46,48,49,50,60], 4 mixed-methods studies [51,53,54,55] and 1 qualitative study [58] (Table 1A). Of the total 24 studies, 14 studies showed a positive association between education and dietary practice, dietary diversity and nutrition knowledge (Table 3A). This indicates that the higher the level of education the better the dietary behaviour of pregnant women. Two studies showed that higher education levels are associated with lower adherence to food taboos and vice versa [40,44], with pregnant women without formal education being 1.97 times more likely to follow dietary restrictions related to food taboos compared to pregnant women with formal education [AOR: 1.97, 95% (CI: 1.58–4.49)]. Six studies reported that education was not associated with dietary diversity [38,55,60], dietary practice [54], nutrition practice [46] and food consumption [29].

Age: Age was examined in twelve quantitative studies [4,8,27,29,39,40,44,46,47,49,50,60], three mixed-methods studies [51,54,55] and one qualitative study [58] (Table 2A). Eleven studies found no association between age and dietary practice [27,60], dietary diversity [4,8,39,49], nutrition practice [46], food consumption score [29], taboo food practice [40,44] and nutrition knowledge [51] of pregnant women. The remaining four studies found associations between the age of pregnant women and dietary diversity [47], nutrition knowledge [50] and dietary practice [54], with one study showing that with increasing age, nutrition knowledge decreases [AOR: 0.175, 95% (CI: 0.001–3.812)] [50]. This was also observed in another study, with pregnant women below 25 years of age [AOR: 4.649, 95% (CI: 1.404–15.396)], and 25–34 years of age [AOR: 3.624, 95% (CI: 1.315–10.269)] being more likely to have adequate minimum dietary diversity compared to pregnant women above 34 years of age [47]. Similarly, a qualitative study found that older women tend to practice food taboos more often than younger women. Among the reasons discussed are that older women were less educated and had less access to health services compared to younger generations [58]. One mixed-methods study showed the opposite result, with older women being more likely to engage in good dietary practice compared to younger women [54].

Occupation: The occupational status of women was investigated in ten quantitative studies [27,32,35,38,40,44,46,47,49,61] and two mixed-methods studies [52,55]. Four studies found positive associations between the occupational status of pregnant women and dietary diversity [38] and dietary practice [27,35,61] (Table 2A). For example, pregnant women who were employed by the government were found to be seven times [AOR: 7.2, 95% (CI: 3.9–17.09)] more likely to adopt good dietary practices than housewives [27]. Another study revealed that pregnant women who were merchants were more likely to adopt appropriate dietary practices compared to housewives [AOR: 2.07, 95% (CI: 1.071–4.016)] [35].

Own monthly income: This factor was studied in only one quantitative [60] and one mixed-methods study [53]. Pregnant women’s own monthly income was positively associated with their dietary diversity, showing that women who had a monthly income were more than two times more likely to have a diversified diet than pregnant women who had no monthly income [AOR = 2.3, 95% (CI: 1.12–4.44)] [60]. Supporting this finding, a mixed- methods study reported that an FGD discussant who was pregnant reported that she had her own small business and regular income that enable her to have a diversified diet not only for herself but also her whole family [53].

Care for women

In the context of care for women, we identified 23 factors at the intrapersonal level for the sublevels physical and biological well-being (*n* = 12/23), cognition (*n* = 4/23) and eating behaviour of pregnant women (*n* = 7/23).

Physical and biological well-being: This was the most frequently investigated sublevel, with 11 factors identified. These include pregnancy complications [52,58]; history of illness [32,33,41,46]; gestational age [29,33,45,60]; gravida (number of pregnancies) [4,40,46,49,51,60,61]; parity (number of times giving birth at a gestational age) [40]; gap between pregnancies [27,48,51]; numbers of lifetime live births [48,49], stillbirths/miscarriages [29], abortions [29] and nutritional status [4] (Table 2A). Four factors, namely, pregnancy complications [51,52], history of illness [33,41], gestational age [33] and gravida [46] showed associations with the dietary behaviour of pregnant women. For example, two quantitative studies found a positive association between the absence of a medical history and dietary practice [33,41]. Women who were not ill two weeks prior to the date of the survey were less likely to have poor dietary practices [AOR = 0.42, 95% (CI: 0.22–0.80] [33], with the other study revealing similar results [AOR = 1.73, 95% (CI: 1.17–2.56)] [41]. Another study reported that women who had been pregnant two or more times were twice as likely to have good nutrition knowledge than women who were pregnant for the first time [AOR: 2.175, 95% (CI: 1.034–4.573)] [46]. In addition, a qualitative study showed that pregnancy complications, such as nausea, vomiting and gastritis, were reported to negatively influence the dietary practice of pregnant women [52].

Cognition: At this sublevel, we identified the factors of attitude and dietary/nutrition knowledge.

Attitude: Attitude was examined by nine quantitative [28,33,34,35,39,40,46,48,61], two mixed-methods [20,53] and one qualitative study [57]. Attitude referred to either a favourable or unfavourable belief or perception of pregnant women towards a health or nutrition topic or an ideal or desired food-related practice. Of the nine quantitative studies that assessed the relationship between attitude and dietary behaviour, six studies showed positive associations between pregnant women’s favourable attitudes towards dietary practice and their actual dietary practice [28,48], frequency of consumption [34], absence of taboo food practice [33,40] and nutrition knowledge [46]. In addition to positive attitudes, some studies also investigated the influence of unfavourable attitudes on dietary behaviour. For example, the qualitative study showed that pregnant women were not interested in preparing and eating a varied diet due to negative attitudes towards food preparation [57]. A quantitative study showed that pregnant women adopted personal beliefs and attitudes about unhealthy eating due to social influence and traditional beliefs and adhered to taboo food practices because they believed that some foods could lead to health problems for the foetus and themselves [57].

Dietary/nutrition knowledge: This refers to accumulated individual knowledge about diets/nutrition. The majority of the studies found positive associations between dietary/nutrition knowledge and dietary practice [20,27,28,30,41,48,54,61] and nutrition practice [46], as well as dietary diversity [8,39,42,52]. One study assessed dietary/nutrition knowledge and its influence on taboo food practices, with pregnant women who had poor nutrition knowledge being more than four times [AOR: 4.94 (95%CI: 3.79–8.75)] more likely to follow food restrictions related to taboo food practices [40].

Dietary/nutrition information: This refers to information received during pregnancy. While this factor was added to the intrapersonal level, it is also reflected at the community level. Twelve studies assessed the influence of dietary/nutrition information during pregnancy on dietary behaviour, with eleven studies identifying positive associations, specifically with the dietary diversity of pregnant women [4,31,42,45,47,49] and dietary practice [28,54,61]. Only one study found no association between dietary/nutrition information and the dietary practice of pregnant women [48]. One mixed-methods study investigated the association between dietary/nutrition information and nutrition knowledge [51], revealing that women who received dietary/nutrition information during pregnancy were more than three times more likely to have good nutrition knowledge [AOR:3.6, 95% (CI: 2.033–6.352)] [51].

Eating behaviour: Eating behaviour at the intrapersonal level comprises seven factors, including meal frequency [8,34,38,39,42,49,50,54,55,60], additional meals during pregnancy [27,37], skipping meals [27,52], food cravings [27,34,52,54], food aversion [35], consumption of animal-sourced foods (ASFs) [34] and khat chewing [35].

Meal frequency: Meal frequency was the most investigated factor at the sublevel of eating behaviour. Five quantitative studies found positive associations between meal frequency and the dietary diversity of pregnant women [8,38,42,49,60]. One of these studies found that increasing meal frequency (one additional meal) increased the likelihood of eating a varied diet by 1.5 times [AOR: 1.5, 95% (CI: 1.04, 2.07)] [60]. In contrast, another study showed that pregnant mothers who received an additional meal were 1.62 times more likely to avoid meals than mothers who did not receive an additional meal [37], suggesting that food avoidance may be due to greater choice. Two quantitative studies showed no association between food consumption and knowledge about a balanced diet [34,50] (Table 2A). Two mixed-methods studies assessed the relationship between meal frequency with dietary diversity and dietary practice of pregnant women and found no association [54,55].

Food cravings: Four studies examined the influence of food cravings on dietary behaviour of pregnant women. Two of these were mixed-methods studies assessing food cravings with dietary practice and dietary diversity, reporting no association [52,54]. Two quantitative studies assessed food cravings with the dietary practice [27] and food consumption frequency [34] of pregnant women, with one study showing that pregnant women who did not have cravings were twice as likely [AOR: 2.07, 95% (CI: 1.41–5.5)] to have good dietary practices compared to those with cravings [27].

Additional meals during pregnancy: Two quantitative studies assessed the association between an additional meal during pregnancy and dietary practice and food aversion. Pregnant women who consumed an extra meal were four times more likely [AOR: 4.7, 95% (CI: 1.6–10.3)] to have adequate dietary practices than women who did not have an additional meal [27]. The other study found that pregnant women who had an additional meal were more likely [AOR: 1.6, 95% (CI: 1.4–1.83)] to avoid food than women who did not have an additional meal [37].

Chewing khat: Khat is a green leaf containing the active ingredient cathinone, causing mild to moderate psychological dependence [62]. In some parts of Ethiopia, especially in the central and eastern parts of Muslim and rural communities, it is considered a stimulant and traditional practice [62]. In this review, we found only one study that investigated the influence of chewing khat on the dietary behaviour of pregnant women, showing that chewing khat resulted in inadequate dietary practices compared to not chewing khat [AOR = 0.58, 95% (CI = 0.37–0.90)] [35].

#### 3.3.2. Interpersonal Level

The interpersonal level is divided into the following sublevels: household socio-economic and -demographic statuses, household access to land and farming and gender roles and responsibilities. In total, we found 21 factors at this level. Most of the factors assessed were related to socio-economic and -demographic statuses (*n* = 12/21), followed by gender roles and responsibilities (*n* = 6/21) and household access to land and farming (*n* = 3/21) (Table 3B).

The sublevel household socio-economic and -demographic statuses include household monthly income, wealth index, household food security, husband’s education, husband’s occupation, household size and place of residence.

Household monthly income: This was assessed in 18 studies, including 14 quantitative studies [27,28,30,31,32,38,39,41,42,45,46,48,50,61] and four mixed-methods studies [51,52,54,55]. Of the 14 quantitative studies, eleven studies and all four mixed-methods studies identified positive associations between the household monthly income and dietary practice [28,30,41,48,54,61], dietary diversity [31,32,39,42,45,52,55], nutrition practice and nutrition knowledge [46,51] of pregnant women. Three quantitative studies reported no association between household monthly income and the dietary practice [27], dietary diversity [38] and nutrition knowledge [50] of pregnant women.

Wealth index: Ten studies examined the association between wealth index and the dietary behaviour of pregnant women [4,8,20,29,34,40,49,53,58,60]. Five quantitative studies and one mixed-methods study found that a higher household wealth index was related to a higher dietary diversity score [4,49,53,60], higher food frequency [34] and lower adherence to food taboo practices [40] among pregnant women. Pregnant women with a low wealth index were 2.26 times more likely to follow food taboos than pregnant women who had a high wealth index [AOR = 2.26 95% (CI: 1.17–4.35)] [40]. One study indicated that the likelihood of adequate dietary diversity among rich pregnant women was more than two times higher than for poor pregnant women [AOR = 2.3, 95% (CI 1.04–5.26)] [60]. One quantitative study showed that pregnant women in households with a poor/low wealth index were more likely to have an unacceptable food consumption score [34]. Two quantitative and one mixed-methods study reported that there was no association between wealth index and the dietary diversity [8], dietary practice [20] and food consumption score [29] of pregnant women.

Household food security: Another commonly studied factor related to the economic aspects of pregnant women was household food security. Six quantitative [8,38,39,43,49,60] and three mixed-methods studies [20,52,53] investigated the association between household food security and the dietary behaviour of pregnant women. Out of these nine studies, six studies identified positive associations. This means that pregnant women in food-secure households were more likely to have diversified diets, compared to pregnant women in food-insecure households [8,20,38,43,52,53]. One study in an urban area showed that pregnant women in severely food-insecure households were more than three times less likely to have adequate diversified diets [AOR: 3.66, 95% (CI: 1.29–10.39)] [43], while another study revealed that pregnant women who lived in food-secure households were more than three times more likely to have an adequately diversified diet than pregnant women in food-insecure households [AOR: 3.85, 95% (CI: 2.12–6.97)] [38]. Three quantitative studies found no association between household food security and the dietary diversity of pregnant women [39,49,60].

Husband’s education: This was assessed in 12 studies, including nine quantitative [4,30,31,35,37,46,48,49,60] and three mixed-methods studies [51,52,54]. Five of the quantitative studies [4,30,46,48,60] and two of the mixed-methods studies [51,54] showed no association between a husband’s level of education and the dietary diversity [4,49,60], dietary practice [30,48,54] or nutrition knowledge [46,51] of pregnant women, while three quantitative studies [31,35,37] and one mixed-methods study [52] found a positive association between a husband’s level of education and the dietary diversity [31,52], dietary practice [35] and lack of food aversions [37] of pregnant women. One study revealed that pregnant women whose husbands had a college degree or higher were two times more likely to have adequate dietary diversity than their counterparts [AOR = 2.45, 95% (CI: 1.20–9.57)] [31]. Another study reported that pregnant women of uneducated husbands were more likely to avoid foods compared to pregnant women whose husbands were educated [AOR = 1.537, 95% (CI: 1.356–1.811)] [37].

Husband’s occupation: This was examined in six quantitative [8,29,31,46,48,60] and two mixed-methods studies [52,54]. Five quantitative studies [8,29,46,48,60] and one mixed-methods study [52] showed no association between a husbands’ occupation and the dietary diversity [8,52], dietary practice [48,60], food consumption score [29] and nutrition knowledge [46] of pregnant women. However, two quantitative [31,46] and one mixed-methods study [54] identified a positive association between a husband’s occupation and the dietary practice [31,54] and nutrition practice [46] of pregnant women. One study found that women whose husbands were employed by the public sector were four times more likely to have adequate dietary practices than women whose husbands were daily labourers [AOR = 4, 95% (CI: 2.18–7.21)] [31]. This was observed in another study that found that pregnant women whose husbands were daily labourers were less likely to have an adequate dietary practice [AOR = 0.058, 95% (CI: 0.01–0.72)] than women whose husbands were regularly employed [54].

Household size: This was assessed by seven quantitative [30,32,36,39,43,60,61] and one mixed-methods study [54]. Three of the quantitative studies and one mixed-methods study found no association between household size and pregnant women’s dietary diversity [32,39,60] and dietary practice [54]. Three quantitative studies found a negative association between household size and the dietary practice [30,43,61] of pregnant women. This shows that as household size increases, pregnant women are less likely to engage in good dietary practices, while as household size decreases, they are more likely to engage in good dietary practices. One study showed the variation in household size and its relationship to women’s dietary practices. Pregnant women who lived in small households (1–3 household members) were more than five times [AOR: 5.66 95% (CI: 2.03–15.83)] more likely and pregnant women who lived in medium-sized households (4–6 household members) were only more than two times [AOR: 2.84 95% (CI: 1.05–7.67)] more likely to engage in optimal dietary practices compared to pregnant women who lived in larger households (≥7 household members) [30]. Another study found that pregnant women who lived in households with more than five members were ten times more likely to have suboptimal dietary practices compared to women who lived in households of ≤5 members [AOR: 10.1 95% (CI: 7.14–19.2)] [43].

Place of residence: Seven quantitative [29,30,36,38,40,49,61] and two mixed-methods studies [52,55] examined the association between place of residence and the dietary behaviour of pregnant women. While five studies found no association between place of residence and the dietary diversity [38,49,55], taboo food practices [40] or dietary practices [30] of pregnant women, four studies found associations between place of residence and dietary behaviour. One quantitative study [29] and one mixed-methods study [52] found that being a rural dweller was associated with inadequate dietary diversity [AOR: 1.91, 95% (CI: 1.01–3.62)] and unacceptable food consumption scores [AOR = 4.594, 95% (CI: 1.871–11.283)] compared to women who lived in urban areas. Similarly, another study showed that pregnant women who lived in urban areas were more than two times more likely to have good dietary practice than women who lived in rural areas [AOR = 2.64, 95% (CI: 1.18–5.92)] [61]. In contrast to the findings above, one quantitative study found that pregnant women who lived in rural areas were more than three times more likely to have adequate dietary diversity than pregnant women who lived in urban areas [AOR: 3.72, 95% (CI: 2.22–6.20)] [36].

Household access to land and farming

This sublevel includes having a home garden and diversity of vegetable and crop production (e.g., vegetable, sweet potato, and coffee), as well as land ownership on the dietary behaviour of pregnant women.

Having a home garden: Two quantitative studies assessed the influence of having a home garden and its association with the dietary diversity of pregnant women [8,36]. One of these studies found that pregnant women who lived in households with a home garden were four times more likely to have adequate dietary diversity [AOR = 4.26, 95% (CI (2.08–8.70)] compared to pregnant women who had no home garden [36]. The second study found no association [8].

Vegetable and crop production: This factor was assessed in one quantitative study [37] and two mixed-methods studies [20,58]. In the quantitative study, a negative association was found between the cultivation of sweet potatoes, potatoes and coffee and the dietary diversity of pregnant women [37]. Specifically, the results showed that pregnant women from households where sweet potato [AOR = 1.332, 95% (CI: 1.221–1.498)], potato [AOR = 1.599; (CI:1.404–1.887)] and coffee [AOR = 2.873; (CI:1.453–5.678)] were grown were more likely to avoid foods than pregnant women in households that did not grow these agriculture products [37]. In contrast, another study that assessed the relevance of edible crop production and vegetable production found positive associations with the dietary diversity of pregnant women. The results show that rural women who lived in households/farms that produced vegetables [AOR = 1.50, 95% (CI:1.1–2.2)] and edible crops [AOR = 2.00, 95% (CI:1.2–3.2)] were more likely to have adequate dietary diversity compared to their counterparts [20].

Ownership of land: Ownership of farm land was assessed in only two quantitative studies, with one of the studies finding a positive association with the food consumption of pregnant women [34], while the other study identified no association between ownership of land and dietary diversity [32].

Care for women related to gender roles and responsibilities was the least studied sublevel at the interpersonal level, including three quantitative [27,32,39] and two mixed-methods studies [20,52]. Six factors were identified including the following: family support, women’s decision making, workload of women, husband’s support, gender roles within household and participation in food shopping.

Family support, women’s decision making in the household and women’s workload in the household: One mixed-methods study assessed the influence of these factors [20]. No association was found between family support at the household level, as well as women’s decision making in a household, and the dietary diversity of pregnant women [20]. However, the study found that pregnant women’s workload had an influence on their dietary diversity [20]. The authors found that pregnant women were heavily burdened by agricultural work and multiple household tasks. For this reason, the women did not eat their meals on time and were also unable to prepare a variety of foods for themselves [20].

Husband’s support: Four quantitative studies assessed the husband’s support [27,32,39,60], with two studies showing a positive association with the dietary diversity of pregnant women [32,60]. Rural pregnant women who received emotional support from their husbands were four times more likely [AOR: 4.0, 95% (CI: 1.16–13.7)] to have adequate dietary diversity than women who did not receive emotional support [60]. One of these studies showed that when the husband was involved in pregnancy matters, women were able to achieve better spacing of pregnancies and also had better nutritional status [60], while the other study [32] did not state what kind of support pregnant women received from their husbands.

Gender roles within household: This was only investigated in one mixed-methods study [52] showing that burdening women with many tasks and responsibilities (i.e., food preparation and related chores) had a negative influence on the dietary practice of pregnant women. One key informant reported that “cultural practices in most rural communities do not pay attention to women. Women are seen as providers for their husbands, so they prepare nutritious food for the husband. The husbands also expect to be served first. This practice will not change even during pregnancy” (page 110) [52].

Participation in food shopping: A quantitative study found a positive association between participation of women in food shopping and the dietary diversity of pregnant women, with pregnant women being 2.5 times more likely to have adequate dietary diversity than women who did not participate [AOR = 2.54, 95% (CI: 3.27–9.83)] [32].

#### 3.3.3. Community Level

This level consists of three sublevels: health and sanitation environment; food environment; and social and cultural norms. Altogether 15 studies explored factors at the community level (Table 3C). Care for women at this level refers to the health and sanitation environment sublevel.

The sublevel health and sanitation environment includes the factors health professionals’ knowledge gap and negligence, ANC visit, source of nutrition information related to health services and source of water and availability of latrines related to sanitation facilities.

ANC visit: This term refers to access to and use of health care during pregnancy, was the most commonly examined factor in the nine studies, including eight quantitative [34,35,40,45,48,49,50,60] and one mixed-methods study [55]. Six studies found a positive association between ANC visit and the dietary diversity [55], dietary practice [45,48], food consumption score [34], knowledge of a balanced diet [50], and nonadherence to taboo food practices [40] of pregnant women. One study reported that pregnant women who had ANC visits were three times [AOR = 3.125, 95%, (CI:1.178–8.291)] more likely to have knowledge of a balanced diet than women who had no ANC visits [50]. In addition, one study showed that pregnant women who had no ANC visits were six times [AOR = 6.16 (95% (CI: 4.99–10.13)] more likely to observe taboo food practices than women who had ANC visits [40]. Three quantitative studies showed no association between ANC visits and dietary diversity [49,60] and dietary practice [35].

Source of information: In a quantitative study conducted in the Oromia region, this factor was assessed in relation to the receipt of dietary information [36]. This study showed that pregnant women who received dietary information from a health professional were five times [AOR = 5.26, 95% (CI (1.60–17.36)] more likely to have adequate dietary diversity than women who received information from neighbours, TV, or the radio [36].

Health professional’s knowledge gap and negligence: A qualitative outcome of the mixed- methods study was the focus group discussions with mothers, husbands and health care professionals and the key informant interviews with pregnant women, health care professionals, nutritionists and community leaders who reported that there was a knowledge gap and negligence among health care professionals, especially nurses and health extension workers, in providing appropriate nutritional advice to pregnant women. This led to inappropriate dietary practices among pregnant women [20].

Source of water: Sources of water in relation to dietary behaviour was assessed in two quantitative studies [35,36]. While one study found no association [35], one study showed positive associations and reported that pregnant women who used protected sources ofwater were twelve times [AOR = 12.49, 95% CI (6.01–25.96)] more likely to have adequate dietary diversity than women who used unprotected sources of water [36].

Availability of latrine: This factor was assessed in two quantitative studies [36,38]. One study found no association between the presence of latrines and the dietary diversity of pregnant women [38]. Another study revealed that there was a positive association showing that pregnant women who had a latrine were six times more likely [AOR = 6.01, 95% CI (2.90–8.84)] to have adequate dietary diversity than women who had no latrine [36].

Food environment

Food environment is the physical interface where consumers interact with the wider food system. In this review, we found only three factors related to the food environment. These include market availability, seasonality of production and agro-ecological conditions identified by one qualitative study [58].

Seasonality of production: In a qualitative study conducted in a rural area of the Oromo region, seasonality of production was found to influence the diversity of pregnant women’s diets [58]. Pregnant women reported that ‘’we (pregnant women) eat what we get at home. We mainly produce maize, barley and beans. We eat injera and shiro [local sauce prepared from beans/peas], but during “belg” (short rainy season) and “kiremt” [main rainy season], we also harvest and consume potato, cabbage and other vegetable’’ (page 3) [58].

Availability of production/market availability: The abovementioned qualitative study also identified the availability of food’s, especially of vegetables and fruits, influence on the dietary diversity of pregnant women. One key informant (a pregnant woman) reported that due to the low production levels of vegetable and fruits there was limited availability of vegetables and fruits in the market, which restrains their consumption of diverse diets [58].

Agro-ecological conditions: Agricultural production depends on agro-ecological conditions, which are partly determined by the differences in altitude between the study areas i.e., weather conditions and or soil characteristics. In the lowlands and highlands, there are many different agriculture products that determine food consumption of pregnant women in the area [58]. Lowland and midland settlers, mainly produce teff, maize, sorghum and, to some extent, wheat, while highlanders produce barley, wheat and sometimes oats, and food consumption is largely determined by what is produced [58].

Cultural and social norms

Four factors were identified at the sublevel of cultural and social norms. Social capital was assessed by a quantitative study [60], and taboo food practices were assessed by five studies including one qualitative [58], three mixed-methods studies [20,52,55] and one quantitative study [35]. Cultural beliefs and social context (e.g., pressure from relatives and friends) that influence the adherence to food taboo were examined by five studies including three qualitative [56,57,58] and two mixed-methods studies [52,55] (Table 3C).

Social capital: In a quantitative study, social capital was assessed based on the number of social networks in which a woman was involved [60]. Pregnant women who participated in women’s social networks like “edir”, (i.e., formal/informal association for financial and psychological support of members of the association) and “ekub” (i.e., formal/informal association for saving and financial support of all members of the association) were more than seven times more likely to have adequate dietary diversity than women who were not part of social networks (AOR = 7.8, (95% CI: 1.02, 59.19)) [60].

Taboo food practice: Taboo food practices and their influence on the dietary behaviour of pregnant women was examined in five studies. A mixed-methods study showed that pregnant women who avoid certain foods, such as yogurt, eggs, banana, linseed and chicken meat, due to cultural beliefs, had inadequate dietary diversity (AOR: 3.05, 95% CI: 1.49, 6.25) [52]. In addition, a quantitative study showed that pregnant women who reported restricting food intake during pregnancy had lower levels of appropriate dietary practice (APR = 0.36, 95% CI = 0.20–0.65) [35]. 

Similarly, a qualitative study reported that pregnant women had poor dietary practice due to adherence to food taboos, such as avoidance of green leafy vegetables, dairy products and some fruits [58]. Moreover, a mixed-methods study showed that cultural prohibition of food affects dietary practice of pregnant women. Key informants and focus group participants, including pregnant women, husbands, health workers and nutritionists, reported the negative consequences of cultural restrictions on maternal nutrition [20]. Pregnant women in the study area were advised by the community to reduce the frequency of meals and to avoid some selected foodstuffs (linseed, pumpkin and chickpea) during pregnancy that affected their dietary practice [20]. Only one mixed-methods study found no association between taboo food practice and dietary diversity of pregnant women [55].

In this review, taboo food practice was identified as one factor that affects the dietary diversity and dietary practice of pregnant women, as presented in the above paragraph. Moreover, we identified five studies that were investigated enabling determinants of taboo food practice among pregnant women [52,55,56,57,58].

#### 3.3.4. Institutional Level

This level contained the following three sublevels: governance and social actions, social and cultural norms and resources (Table 3D). Care for women at this level refers to good governance with regard to political, financial, social, public and private sector actions that enhance the right to adequate and healthy diets for pregnant women. In total, we identified five factors assessed by five quantitative [4,29,40,44,50] and three mixed-methods studies [20,53,55].

Governance and social actions: At this sublevel, we identified only two factors including women’s empowerment [53] and productive safety net programs (PSNPs) [4].

Women’s empowerment: This was only examined in one study, which found that pregnant women with higher levels of female empowerment (i.e., women’s involvement in household decision making, membership in a community group, cash earning, ownership of household/land and education) were three times more likely [AOR = 3.497, 95% CI: 2.301–5.315] to have good dietary diversity than their counterparts [53].

Productive Safety Net Program PSNP: The results of a quantitative study showed that pregnant women who were recipients of productive safety net programs (PSNP) had lower dietary diversity scores than pregnant women who did not benefit from the program [4]. PSNPs target vulnerable social groups, prioritising women and children due to food insecurity and low wealth index. The author recommended that strengthening sustainable income-generating activities and saving strategies to improve the wealth status of pregnant women could improve the dietary diversity of pregnant women.

Social and cultural norms

At the sublevel of social and cultural norms, three factors, including ethnicity [40,50], religion [29,44,55] and fasting [20,44,50], were examined in six studies (Table 3D).

Religion: Two quantitative [29,44] and one mixed-methods study [55] assessed religion in relation to the dietary behaviour of pregnant women. While two studies found no association between religion and dietary diversity [55] and between religion and taboo food practices of pregnant women [44]. One quantitative study found a positive association between religion and the frequency of food consumption of pregnant women showing that pregnant women who were Muslim, Protestant and Catholic followers were nearly 92.7% less likely to have unacceptable food consumption score (i.e., infrequent food consumption) as compared to those who were Orthodox Christians [29].

Fasting: Two mixed-methods studies showed that fasting in the Orthodox Christian religion increased inappropriate dietary practices and dietary diversity of pregnant women [20,53]. In one of these studies, group discussions revealed that “pregnant women abstain from eating animal products, and they do not take breakfast during fasting days. This prevents them from taking an adequate diet” (page 8) [20]. In a quantitative study, no association was found between fasting and perception of a balanced diet of pregnant women [50].

Ethnicity: Two quantitative studies assessed the association between ethnicity and taboo food practices of pregnant women [40,50]. While one study did not find an association between ethnicity and the taboo food practices of pregnant women [40], one study reported that pregnant women who belonged to the Oromo ethnic group were fourteen times more likely to observe food taboos than women who belonged to the Wolayta ethnic group (AOR = 14.988, (95% CI: 1.681–133.644)) [50].

Resources

We added the sublevel of resources to the institutional level of the socio-ecological framework. Resources in the sense of the UNICEF framework refer to sufficient environmental, financial, social and human resources. In this review, we found no study that assessed any factor related to resources and its influence on the dietary behaviour of pregnant women.

## 4. Discussion

In this mapping review, we identified and synthesised 68 factors across four levels of the socio-ecological framework in the context of dietary behaviour of pregnant women in Ethiopia. This review underscores the importance of care for women as a critical aspect for their dietary behaviour and maternal health [13]. Accordingly, we integrated two conceptual frameworks, the socio-ecological framework and the UNICEF framework of maternal and child nutrition, to highlight the aspect of care and its importance at the intrapersonal, interpersonal, community and institutional levels. Most of the evidence in our review was found for socio-demographic and -economic factors at the intrapersonal and interpersonal levels, while fewer factors were identified at the other levels of influence, including at the community and institutional levels. These findings are in line with previous reviews in Africa by Osei Kwasi et al. (2020) [21], Yiga et al. (2021) [17], Stadlmayr et al. (2023) [63] and Trübswasser et al. (2021) in LMICs [64]. Despite the research gap on factors other than socio-demographic and -economic factors, we found important evidence at other levels of influence that underscore the need for holistic, systemic approaches to improve the dietary behaviour and, in turn, the health of pregnant women and their children. This will be discussed in the following section.

Integration of two conceptual frameworks

Care for women at the intrapersonal level emphasises the central role of women for their own healthy dietary behaviour. However, it is important to note that related actions and behaviours depend, to a large extent, on available resources and on norms that determine access to resources and decision making for women. In the UNICEF framework, this is shown as enabling determinants, consisting of (1) sufficient resources, including environmental, financial, social and human resources that enable women’s (and children’s) right to nutrition—reflected in the socio-ecological framework at the institutional level; and (2) norms, referring to positive cultural norms and actions that enable women’s (and children’s) right to nutrition. In the socio-ecological framework, social and cultural norms are included at the following different levels: institutional, setting the broader frame; community, shown as cultural beliefs that influence food practices; interpersonal/household, reflected as gender roles and responsibilities. Therefore, they impact on actions and behaviours at all levels, especially at the intrapersonal level. This is why we included the aspect care for women, as highlighted in the UNICEF framework, at each level of our adapted socio-ecological framework. Sufficient resources are partly reflected in the sublevel economic status of women at the intrapersonal level, and are also relevant at the interpersonal/household level (socio-economic status of household members) and community level (food environment).

Intrapersonal level

At the intrapersonal level, women’s educational status was the most studied factor, followed by age and occupation, as part of the socio-economic and -demographic sublevels. Evidence for the educational status of women showed that women who have higher levels of education are more likely to have healthier dietary behaviours. Educating women translates to educating the whole family, given their central role in household responsibilities, including domestic activities and taking care of children. A study across 116 LMICs (over a period of 42 years) investigated the relationship between two indicators of women’s control over their lives, as follows: the number of girls attending secondary school and the ratio of female-to-male life expectancies. Improving these two indicators reduced stunting in children by 32% [65]. The promotion of gender equality in education is a measure to improve care for children and women in terms of their rights, freedom and privilege of access to food and good nutrition [13]. 

Limited evidence exists on women’s own income. Surprisingly, in our review, out of 37 studies only two studies assessed the association between women’s economic status and dietary behaviour, showing that women with own income, savings account and mobile phones were more likely to have adequate dietary diversity. The reason for the scarcity of studies could be due to the fact that in most cases the husband or male partner is supposedly still the main contributor to household income, having the financial power and being in charge of decision making, due to patriarchal structures [59,66]. Knowledge about nutrition, as well as food attitudes, were two frequently studied factors under the sublevel cognition and were found to positively influence dietary behaviour. In line with our findings other reviews revealed that women who have good knowledge about nutrition or healthy diets were more likely to have healthier dietary behaviour [17,21,22,67]. Nevertheless, nutrition knowledge of pregnant women is interlinked with community level factors. While several studies assessed whether receiving nutrition information during pregnancy influences dietary behaviour, only one study assessed the influence of source of nutrition information during pregnancy [36] at the community level. The study showed that women who received nutrition information from a health professional were more likely to have adequate dietary diversity than women who received information from other sources [36]. This means that availability of and access to health services that provide nutrition information during pregnancy is of paramount importance. The responsibility of gaining access to such information cannot be placed solely on the individual mother. While most factors were identified under the sublevel of physical and biological well-being, the evidence is scattered. In addition, we expected to find psychological factors in this review, such as being emotional, feeling stressed and experiencing depression and anxiety, which influence pregnant women’s eating behaviour [67,68,69]. Moreover, under the sublevel eating behaviour, chewing khat was the least studied in this review. Only one study assessed chewing khat during pregnancy in relation to dietary practice of pregnant women showing that pregnant women who chew khat had inadequate dietary practices [35]. The practice of chewing khat is common in some Ethiopian communities [62]. A study reported that avoiding some social practices can promote healthier diets; meanwhile, it can lead to feelings of isolation in the community [67]. Therefore, more research is needed to understand the influence of chewing khat on pregnant women’s dietary behaviour.

Interpersonal level factors

At the interpersonal level, the most consistent evidence was found for socio-economic factors, showing that the dietary behaviour of pregnant women improved with higher levels of household monthly income, as well as with higher wealth index of households. This finding is not surprising for the study area and population group and is consistent with other studies [17,21,70], as low-socio-economic status is a challenge for healthy dietary behaviour, especially in lower- and middle-income countries. Factors related to household access to land and farming were assessed by only two studies, despite the fact that this is a crucial condition for ensuring adequate access to a diversified diet, addressing underlying structural inequalities [71]. For example, land ownership for women can reduce gender inequalities and give women more control over resources, strengthen their economic independence and decision-making power, and increase their social status and influence in society [72]. The two studies identified present contradictory results. One study found that pregnant women with access to farmland in the household had better food consumption scores [34], while the other study found no association between access to farmland and dietary diversity of pregnant women [32]. In both studies, intrahousehold decision making and gender disparities within households were not discussed. It is therefore difficult to assume the reasons for the contradictory results. Clearly, more research is required to determine the influence of these factors on women’s dietary behaviour. Further, different from what we would have expected, few studies identified factors related to gender roles and responsibilities, here including family support, women’s decision making, women’s workload, husband’s support, gender roles within households and participation in food purchasing. This is despite the fact that these are core aspect of care for women and linked to enhancing dietary behaviour. The most consistent evidence was found for pregnant women having their husbands’ emotional support with pregnancy matters, compared to not having this support, and the resulting positive or negative impacts on dietary behaviour. These findings highlight that intrahousehold dynamics must be placed at the centre of efforts to ensure healthy diets for pregnant women and their unborn children, instead of focusing on pregnant women in isolation. In low- and middle-income countries, including Ethiopia, men are commonly still regarded as head of the family, being responsible for generating financial income and having decision-making power in the household [59,70,73,74,75]. Therefore, supportive partners play an important role for the well-being of pregnant women. Studies identified the importance of husbands’ support during pregnancy for maternal nutrition in Bangladesh [76] and Tanzania [71]. The World Health Organization (WHO) reported the significance of emotional support of the family as one of the indicators of quality of care for women and newborn children to promote a healthy life. In particular, support from the male partner during pregnancy, childbirth, and the post-partum period is an effective strategy to improve both maternal and child health outcomes [14]. Moreover, women’s participation in decisions at household level should be considered an essential aspect of gender roles and responsibilities, particularly concerning adequate care for women. Studies in Ethiopia [77,78], Ghana [79] and India [75] showed that women’s participation in household decisions improved their dietary diversity. In this review, we found only one study on women’s participation in household decisions, with no association to their dietary practice [20]. On the other hand, as elaborated in Section 3, one study found a positive association between women’s participation in food purchase and dietary diversity [32]. In line with this, a study in Nigeria revealed that in male-headed households, decision-making power lies with husbands and pregnant women who do not participate in food purchase have poor dietary practices [70].

Community level

Social and cultural norms and the health and sanitation environment, as well as the food environment, play important roles in promoting dietary behaviour at the community level. Among these three sublevels, most studies assessed factors related to the health and sanitation environment, while the food environment and social and cultural norms were rarely investigated in the studies included in our review. Antenatal care (ANC) visits was the most frequently studied factor. Six out of nine studies found an association between ANC visits with pregnant women´s dietary behaviour. For instance, pregnant women attending more often ANC visits had better dietary diversity [45,55]. A study in Somalia confirmed that with increasing ANC visits dietary diversity of pregnant women increased [80]. This emphasises the importance of routine ANC visits for healthy dietary behaviour and health of pregnant women. The World Health Organization (WHO) also highlights the importance of ANC visits to reduce maternal and child mortality, particularly in low- and middle-income countries. In addition, WHO recommends health system-level interventions to improve the utilisation and quality of ANCs [81]. Regarding the sanitation environment, only two factors, namely, source of water and availability of latrines, were identified in three studies. One study found that pregnant women who were using protected well water and who had access to latrines were more likely to have diversified diets [36], while a quantitative study revealed that lactating mothers who used protected well water were more likely to have inadequate dietary diversity than when using tap water [82]. UNICEF underlines the significance of the health and sanitation environment for maternal and child nutrition [12]. Access to basic infrastructure, such as clean water, sanitation, and hygiene, is fundamental for a healthy environment [83]. In Ethiopia, less than half (47.3%) of rural households have access to basic improved drinking water that is located within a 30-min roundtrip for collection [84]. Women and girls are mainly responsible for fetching, storing and treating water for the household, while at the same time they are the ones who are affected most by poor access to water, sanitation and hygiene services [83]. One of the standards of quality care for women and their children is an appropriate physical environment, with adequate water, sanitation and energy supplies in health centres [14] and also safe drinking water, sanitation and improved hygiene, as targets of the SDGs that should be achieved by 2030 [85]. Hence, we expected more studies to examine a broader range of health care factors related to infrastructure and health services and their influence on dietary behaviour of pregnant women. However, this review shows that studies on this subject are scarce. With regard to cultural and social norms, we identified the factors food taboo practices, cultural and social beliefs and social capital. Consistent with the findings of this review, a previous systematic review also identified that social capital highly influences the dietary practice of pregnant women [86]. In Ethiopia, women build formal or informal social networks, such as women’s edir or ekub for financial and psychological support. Encouraging women to establish social capital/networks is important to combat gender inequality and discrimination, as well as harassment of women, as social networks foster collective agency and social cohesion within communities [87,88]. In addition to the abovementioned factors on cultural and social norms, in our earlier empirical study, we identified that cultural and social norms affect the dietary diversity of women of reproductive age [86]. For instance, by using proverbs and sayings, society establishes eating manners regarding what and how women should consume food, based on misperceptions about women and promoting gender inequality [78].

Institutional level

At the institutional level, we categorised the factors according to the sublevels of governance and social actions in relation to care for women, cultural and social norms and resources. Although governance and social actions at the institutional level influence pregnant women’s dietary behaviours, according to our review, these factors were rarely examined. Women’s empowerment and being a beneficiary of a productive safety net programme (PSNP) in terms of care for women are factors that were significantly associated with the dietary behaviour of pregnant women. The government as a national institution is obliged to take care of the safety and security of its citizens. Only one study found that women who felt empowered were three times more likely to have adequate dietary diversity [53]. This was also confirmed by studies in Ghana and Nepal [79,88]. The government should formulate social welfare policies to enhance healthy and adequate diets for pregnant women. For example, Ethiopia’s Productive Safety Net Programme (PSNP) aims to reduce vulnerability to food insecurity by creating economic opportunities and building resilience to crises through cash transfers, public works and nutrition programmes. However, due to the current emerging political problems in Ethiopia, such as the recent civil war and resulting ongoing violence, people are being displaced from their homes, with women and children being most severely affected, not only regarding food insecurity but also by rape and sexual violence [89]. More studies are needed to understand factors at the institutional level so that appropriate interventions can be developed. At the institutional level, factors related to cultural and social norms were the least frequently assessed. Ethnicity and religion influence dietary behaviour of pregnant women to a large extent. Although there are more than 85 ethnic groups with different religious practices in Ethiopia, our review implies that not enough attention has been given towards understanding cultural and religious aspects. In our earlier empirical study and other studies, fasting was found to negatively affect the dietary practice of women [20,78]. In this review, we did not find any factors related to resources at the institutional level.

## 5. Conclusions

In this review, we identified and mapped factors that influence the dietary behaviour of pregnant women in Ethiopia. We adapted the socio-ecological framework by integrating factors from the literature and the UNICEF conceptual framework on maternal and child nutrition, making the aspect care for women visible. The most consistent evidence was found at the intrapersonal level for socio-demographic and -economic factors, while fewer factors were identified at the other levels. Our innovative conceptual approach reveals a research gap regarding care for women at all levels and identifies neglected key aspects impacting dietary behaviour. This review shows that little is known with regard to socio-cultural and environmental aspects, including socio-cultural norms within households and communities. There is a further gap regarding the influence of various resources at the institutional level. Overall, we identified a focus on the individual responsibility of women, while underlying aspects are still rarely studied. Clearly, more qualitative studies on this subject are needed. Future research should consider the interconnections of factors at all levels of the socio-ecological framework and place emphasis on care for women and healthy environments as fundamental for achieving the adequate nutrition and well-being of women and their children.

## Figures and Tables

**Table 1:** (**A**) Inclusion and exclusion criteria for the selection of studies; (**B**) inclusion and exclusion criteria for the selection of qualitative studies.

(**A**)		
	Inclusion criteria	Exclusion criteria
Population	Pregnant women	Pregnant women with chronic diseases, women, children and other population groups
Outcome	Dietary behaviour (i.e., food choice, eating behaviour and dietary intake/nutrition)	Nutrition status of pregnant women
Setting/context	Ethiopia	Other than Ethiopia
Type of study	Qualitative, quantitative and mixed- methods studies	Review papers, conference abstracts, and records without access to full text
Language	English	Other than English language
Time	2000 to 2022	Studies before and after 2000–2022
(**B**)		
	Inclusion criteria	Exclusion criteria
Setting	Ethiopia	Other than Ethiopia
Perspective	Pregnant women	Children and men
Phenomena of interest	Perceptions related to dietary behaviour, dietary practice, dietary diversity, quality of diet during pregnancy, Perceptions regarding enablers and barriers towards dietary behaviour during pregnancy	
Evaluation	Studies with qualitative study design	Review papers, conference abstracts, records without access to full text
Language	English	Other than English language
Year of publication	2000 to 2022	Studies before and after 2000–2022

**Table 2:** (**A**) Characteristics of the quantitative studies on factors influencing the dietary behaviour of pregnant women; (**B**) characteristics of the mixed-methods studies and qualitative studies on the factors influencing the dietary behaviour of pregnant women.

(**A**)									
**Author**	**Publication Year**	**Region**	**Setting**	**Method of Data Collection**		**Age (Years)**	**Sample Size (n)**	**Sampling**	
Abute et al. [27]	2020	SNNPR	Rural and urban	Structured interviews		18–36	618	Simple random sampling	
Alemayehu et al. [28]	2015	Amhara	Urban	Structured interviews		15–41	574	Cluster random sampling	
Aliwo et al. [4]	2019	Amhara	Rural	Structured interviews		17–≥35	647	Cluster and simple random sampling	
Ambaw et al. [29]	2021	Amhara	Urban	Structured interviews		18–49	422	Systematic random sampling	
Belay et al. [30]	2022	Amhara	Rural and urban	Structured interviews		≤19–≥30	615	Simple random sampling	
Delil et al. [31]	2021	SNNPR	Urban	Structured interviews		<25–≥35	303	Simple random sampling	
Desta et al. [32]	2019	Oromia	Urban	Structured interviews		<25–44	312	Systematic random sampling	
Diddana [33]	2019	Amhara	Urban	Structured interviews		18–35 and above	604	Two-stage sampling	
Fite et al. [34]	2022	Oromia	Rural	Structured interviews		16–36	448	Simple random sampling	
Fite et al. [35]	2022	Oromia	Rural	Structured interviews		16–37	448	Simple random sampling	
Girma et al. [5]	2022	SNNPR	Rural and urban	Structured and semi-structured interviews		16–≥35	566	Systematic random sampling	
Hailu et al. [36]	2019	Oromia	Rural and urban	Structured interviews		<30 and ≥30	413	Systematic random sampling	
Handiso [37]	2015	SNNPR	Rural	Structured interviews		19–49	605	Two-stage cluster sampling	
Jemal et al. [38]	2019	Tigray	Urban	Structured interviews		18–49	412	Systematic sampling	
Kebekde et al. [39]	2022	Addis Ababa	Urban	Structured interviews		<24≥35	320	Simple random sampling	
Mengie et al. [40]	2022	Somali	Rural and urban	Structured interviews		15–>34	636	Cluster sampling technique	
Nana et al. [41]	2018	Amhara	Urban	Structured interviews		19–<35	616	Cluster sampling	
Nigussie et al. [42]	2022	Dire Dawa	Rural and urban	Structured interviews		<19>30	448	Simple random technique	
Shemsu et al. [43]	2020	Oromia	Urban	Semi-structured interviews		15–49	378	Systematic random sampling	
Tale et al. [44]	2020	Tigray	Urban	Semi-structured interviews		>20	332	Stratified random sampling	
Tariku et al. [44]	2022	SNNPR	Rural	Structured interviews		Not stated	367	Cluster sampling	
Tesfa et al. [45]	2021	Addis Ababa	Urban	Structured interviews		18–45	336	Systematic random sampling	
Tenaw et al. [46]	2018	Addis Ababa	Urban	interviews		15–44	322	Systematic sampling techniques	
Tilahun et al. [47]	2021	SNNPR	Rural and urban	Structured and semi-structured interviews		16–≥35	274	Systematic random sampling	
Tsegaye et al. [8]	2020	Oromia	Rural	Structured interviews		≤24–≥35	403	Multistage clustered sampling	
Yalewdeg et al. [48]	2020	SNNPR	Rural	Structured and semi-structured interviews		15–49	351	Lottery method	
Yeneabat et al. [49]	2019	Amhara	Rural and urban	Structured interviews		18–40	834	Multistage sampling technique	
Zepro [50]	2015	Oromia	Rural	Structured interviews		195–<35	295	Systematic sampling	
(**B**)									
**Author**	**Publication Year**	**Region**	**Setting**	**Method of Data Collection**	**Population**	**Age (Years)**	**Sample Size (n)** **Quan. Qual.**		**Sampling**
Daba et al. [51]	2013	Oromia	Rural and urban	Semi-structured questionnaire and focus group discussions	Pregnant women	15–≥44	422	12 Key informants 2 FGDs	Systematic sampling and purposive sampling
Demilew et al. [20]	2020	Amhara	Rural	Structured questionnaires, focus group discussions and interviews	Mothers, husbands, and health professionals	<20–35 Qual. 25 to 55	712	43 Key informants 3 FGDs involving 6–12 participants	Cluster sampling technique and purposive sampling
Geta et al. [52]	2022	SNNPR	Rural and urban	Structured questionnaire	Pregnant women, health extension workers and women’s development army		684	55 Key informants 3 FGDs	Multistage cluster sampling and purposive sampling
Gudeta et al. [53]	2022	SNNPR	Rural and urban	Structured questionnaire	Pregnant mothers, husbands, and health professionals)	<25 and >35	726	40 Key informants 3 FGDs	Multistage cluster sampling and purposive sampling
Tolera et al. [54]	2018	Oromia	Rural	Structured questionnaires, FGD	Pregnant mothers	18–36	343	15 Key informants 2 FGDs	Simple random sampling technique
Wondmeneh [55]	2022	Afar	Rural and urban	Structured questionnaires	Pregnant mothers	≤20–31	241	38 Key informants 6 FGDs	Systematic sampling technique and purposive sampling
Hadush et al. [56]	2017	Afar	Rural and urban	FGDs and in-depth interviews (IDIs)	Pregnant women, lactating mothers and elderly women	21–66		29 Key informants 4 FGD (6 to 8 homogeneous participants	Purposive sampling
Tsegaye et al. [57]	2021	Oromia	Rural	Focus group discussions; in-depth interviews	Health care providers, health extension workers, elders (mothers-in-law), husbands of pregnant women	20–63		79 Key informants 8 FGDs (8–10 participants)	Purposive sampling
Zerfu et al. [58]	2016	Oromia	Rural	Open-ended questions, focus group discussions	Pregnant women and their husbands	23–92		38 Key informants 8 FGDs (8–10 participants)	Purposive sampling

**Table 3:** (**A**): Factors influencing dietary behaviour at the intrapersonal level based on the adapted socio-ecological framework; (**B**) factors influencing the dietary behaviour of pregnant women at the interpersonal level based on the adapted socio-ecological framework; (**C**) factors influencing the dietary behaviour of pregnant women at the community level based on the adapted socio-ecological framework; (**D**) factors influencing the dietary behaviour of pregnant women at the institutional level based on the adapted socio-ecological framework.

(**A**)						
**Intrapersonal Level**	**Sublevel**	**Factors**	**Evidence**			
			**Qualitative and** **Mixed-Methods Studies** **(Qualitative Methods)**	**Quantitative and Mixed-Methods Studies (Quantitative Methods)—No Association**	**Quantitative and Mixed-Methods Study (Quantitative Methods)—** **Association**	**Dietary Behaviour**
	Women’s socio-economic and demographic statuses					
		Mother’s education		[54]	[27,28,30,48]	Dietary practice
			[55]	[38,55,60]	[4,8,31,32,39,42,45,49,53]	Dietary diversity
			[58]		[40,44]	Taboo food practice
					[46,50,51]	Nutrition knowledge
				[46]		Nutrition practice
				[29]		Food consumption
		Mother’s occupation		[32,47,49,52,55]	[38]	Dietary diversity
					[27,35,61]	Dietary practice
				[46]		Nutrition practice
				[46]		Nutrition knowledge
				[40,44]		Taboo food practice
		Own income	[53]		[60]	Dietary diversity
		Marital status		[40]	[44]	Taboo food practice
		Age		[27,60]	[54]	Dietary practice
				[51]	[50]	Nutrition knowledge
				[4,8,39,49]	[47,55]	Dietary diversity
				[46]		Nutrition practice
			[58]	[40,44]		Taboo food practice
				[29]		Food consumption
		Years of marriage		[60]		Dietary diversity
		Mobile phone available			[36]	Dietary diversity
		Bank account		[8]	[36]	Dietary diversity
	Care for women					
	Physical and biological well-being					
		Pregnancy complications (nausea, vomiting and other conditions like gastritis)				
			[52,58]			Dietary diversity
		History of illness		[32]	[33,41]	Dietary practice
				[46]		Nutrition knowledge
		Gestational age (stage of pregnancy)			[33]	Dietary practice
				[45,60]		Dietary diversity
				[29]		Food consumption
		Gravida (number of pregnancies)		[51]	[46]	Nutrition knowledge
				[40]		Taboo food practice
				[60,61]		Dietary practice
				[4,49]		Dietary diversity
		Previous delivery (normal/abnormal)		[46]		Nutrition practice
		Parity (number of times giving birth to a foetus with a gestational age)		[40]		Taboo food practice
		Gap between pregnancy		[27,48]		Dietary practice
				[51]		Nutrition knowledge
		Number of lifetime live births		[49]		Dietary diversity
				[48]		Dietary practice
		Nutrition status		[4]		Dietary diversity
		Still birth		[29]		Food consumption
		Abortion		[29]		Food consumption
	Psychological well-being					
	Cognition					
		Food attitude	[20]	[61]	[20,28,48]	Dietary practice
				[39]	[53]	Dietary diversity
			[57]		[40]	Taboo food practice
					[46]	Nutrition knowledge
				[46]		Nutrition practice
					[34]	Food consumption
		Attitude towards a health or nutrition problem (perceived severity to malnutrition)		[20,33,35]		Dietary practice
		Attitude towards a health or nutrition problem (perceived benefits of good nutritional practice)		[20,33,35]		Dietary practice
		Attitude towards a health or nutrition problem (perceived self-efficacy to control malnutrition)		[20,35]	[33]	Dietary practice
		Attitude towards a health or nutrition problem (intention to eat a balanced diet)		[20]		Dietary practice
		Dietary/nutrition knowledge	[20]		[20,27,28,30,41,48,54,61]	Dietary practice
			[52,53]	[60]	[8,39,42,52]	Dietary diversity
					[40]	Taboo food practice
					[46]	Nutrition practice
				[29]		Food consumption
		Nutrition information during pregnancy			[4,31,42,45,47,49]	Dietary diversity
				[48]	[28,54,61]	Dietary practice
					[51]	Nutrition knowledge
		Health information		[54]		Dietary practice
	Eating behaviour					
		Meal frequency		[55]	[8,38,42,49,60]	Dietary diversity
				[50]		Balanced diet
				[34]		Food consumption
				[54]		Dietary practice
		Additional meal			[27]	Dietary practice
					[37]	Food aversion
		Skipping meal			[52]	Dietary diversity
				[27]		Dietary practice
		Food carving		[54]	[27]	Dietary practice
				[34]		Food consumption
				[52]		Dietary diversity
		Food aversion		[35]		Dietary practice
		Khat chewing			[35]	Dietary practice
		Consumption of ASFs			[34]	Food consumption
(**B**)						
**Interpersonal level**	**Sublevel**	**Factors**	**Evidence**			
			**Qualitative and** **Mixed-Methods Studies** **(Qualitative Methods)**	**Quantitative and Mixed-Methods Studies (Quantitative Methods)—No Association**	**Quantitative and Mixed-Methods Studies (Quantitative Methods)—** **Association**	**Dietary Behaviour**
	Family socio-economic and demographic statuses	Husband’s education		[46,51]		Nutritional knowledge
					[37]	Food aversion
				[4,49,60]	[31,52]	Dietary diversity
				[30,48,54]	[35]	Dietary practice
		Household education			[38]	Dietary diversity
		Husband’s occupation		[29]	[46]	Nutritional practices
				[48,60]	[31,54]	Dietary practice
				[8,52]		Dietary diversity
		Age of husband			[47]	Dietary diversity
		Household monthly income	[54]	[27]	[28,30,41,48,54,61]	Dietary practice
			[52]	[38]	[31,32,39,42,45,52,55]	Dietary diversity
				[50]	[46,51]	Nutrition knowledge
					[46]	Nutrition practice
		Wealth index	[53,58]	[8]	[4,49,53,60]	Dietary diversity
				[20]		Dietary practice
				[29]	[34]	Food consumption score
					[40]	Taboo food practice
		Household food insecurity	[52,53]	[39,49,60]	[8,38,43,52,53]	Dietary diversity
					[20,43]	Dietary practice
		Owning a radio/TV			[41]	Dietary practice
		Livestock ownership			[32]	Dietary diversity
		Place of residence			[29]	Food consumption score
				[38,49,55]	[36,52]	Dietary diversity
				[30]	[61]	Dietary practice
				[40]		Taboo food practice
		Household size		[54]	[30,43,61]	Dietary practice
				[32,39,60]	[36]	Dietary diversity
		Household head		[47]		Dietary diversity
	Household access to land and farming					
		Home garden present		[8]	[36]	Dietary diversity
		Vegetable and crop production (edible crop production)	[58]		[20]	Dietary practice
		Vegetable and crop production (vegetable production)			[20]	Dietary practice
		Vegetable and crop production (growing sweet potato)			[37]	Food aversion
		Vegetable and crop production (growing potato)			[37]	Food aversion
		Vegetable and crop production (growing coffee)			[37]	Food aversion
		Land ownership		[32]		Dietary diversity
					[34]	Food consumption score
	Care for women					
	Gender role and responsibility					
		Work load	[20]			Dietary practice
		Gender role (prioritising husbands for food allocation)	[52]			Dietary diversity
		Husband support		[39]	[32]	Dietary diversity
				[27]		Dietary practice
		Family support		[20]		Dietary practice
		Women’s decision making		[20]		Dietary practice
		Participated in food shopping			[32]	Dietary diversity
(**C**)						
**Community Level**	**Sublevel**	**Factors**	**Evidence**			
			**Qualitative and** **Mixed-Methods Studies** **(Qualitative Methods)**	**Quantitative and Mixed-Methods Studies (Quantitative Methods)—** **No Association**	**Quantitative and Mixed-Methods Studies (Quantitative Methods)—** **Association**	**Dietary Behaviour**
	Care for women					
	Health and sanitation environment					
	Health service	Health professionals’ knowledge gap and negligence	[20]			Dietary practice
		ANC visit		[49,60]	[45,55]	Dietary diversity
				[35]	[48]	Dietary practice
					[34]	Food consumption score
					[40]	Taboo food practice
					[50]	Knowledge about balanced diet
		Source of nutrition information			[36]	Dietary diversity
	Sanitation facilities					
		Source of water			[36]	Dietary diversity
				[35]		Dietary practice
		Availability of latrine		[38]	[36]	Dietary diversity
	Food environment					
		Market availability	[58]			Dietary diversity
		Argo-ecologic conditions	[58]			Dietary practice
		Seasonality of production	[58]			Dietary diversity
	Cultural and social norms					
		Social capital			[60]	Dietary diversity
		Taboo food practice		[55]	[52]	Dietary diversity
			[58]		[20,35]	Dietary practice
		Reason for taboo food practice (to prevent disease)	[55,56,57,58]			Taboo food practice
		Reason for taboo food practice (skin discoloration)	[56]			Taboo food practice
		Reason for taboo food practice (makes the baby large and difficult to delivery)	[52,55,56,57,58]			Taboo food practice
		Reason for taboo food practice (food would plaster onto baby’s head)	[55,57]			Taboo food practice
		Reason for taboo food practice (to prevent abortion)	[52]			Taboo food practice
		Reason for taboo food practice (acceptance of community beliefs, without understanding)	[57]			Taboo food practice
		Reason for taboo food practice—social context (pressure from relatives and friends)	[57]			Taboo food practice
(**D**)						
**Institutional Level**	**Sublevel**	**Factors**	**Evidence**			
			**Qualitative and** **Mixed-Methods Studies** **(Qualitative Methods)**	**Quantitative and Mixed-Methods Studies (Quantitative Methods)—No Association**	**Quantitative and Mixed-Methods Studies (Quantitative Methods)—** **Association**	**Dietary Behaviour**
	Care for women					
	Governance and social actions					
		Women empowerment			[53]	Dietary diversity
		PSNP beneficiary			[4]	Dietary diversity
	Social and cultural norms					
		Ethnicity		[40]	[50]	Taboo food practice
		Religion			[29]	Food consumption score
				[44]		Taboo food practice
				[55]		Dietary diversity
		Fasting	[20]			Dietary practice
			[53]			Dietary diversity
						Taboo food practice
				[50]		Nutrition knowledge
	Resources					
